# Sugar coating autophagy: exploring the links between the inhibition of NGLY1 (N-glycanase 1) and autophagy induction

**DOI:** 10.1080/27694127.2023.2166324

**Published:** 2023-01-16

**Authors:** Holger B. R. Kramer, Sarah Ann Allman

**Affiliations:** a Department of Physiology, Anatomy and Genetics, University of Oxford, UK; b MRC London Institute of Medical Sciences, UK; c Reading School of Pharmacy, University of Reading, UK

**Keywords:** autophagy, autophagy induction, caspase inhibition, ERAD, glycoprotein catabolism, NGLY1, peptide:*N*-glycanase, Z-VAD-fmk

## Abstract

The cytosolic enzyme NGLY1 (N-glycanase 1) is a central mediator of glycoprotein catabolism. The enzyme acts to cleave *N*-linked glycans from modified substrate asparagine residues prior to degradation of misfolded proteins by the proteasome, playing a key and well-conserved role in the ER-associated degradation/ERAD pathway. In a clinical context, NGLY1 disorder represents a rare congenital disorder of deglycosylation where mutations in the *NGLY1* gene result in the loss of enzyme function. Patients with NGLY1 disorder present with a broad and varied array of symptoms, which can include moderate to profound levels of developmental delay, seizures, and complex movement disorders, as well as alacrima. Our recent results highlight a causal link between NGLY1 inhibition and macroautophagy/autophagy induction.

Whereas the relationship between NGLY1 and cellular autophagy processes had not previously been described in the literature, our work [1] shows that both inhibition of NGLY1 by Z-VAD-fmk and similarly by siRNA-mediated NGLY1 knockdown both lead to a robust induction of cellular autophagy in HEK293 cells. In addition, autophagosome immunoprecipitation and mass spectrometry-based proteomics approaches identify comparable autophagosomal protein content following both treatment with Z-VAD-fmk and following NGLY1 knockdown. Furthermore, we were able to show that the observed increase in GFP-LC3-positive puncta (as a measure of increased autophagosome formation) upon NGLY1 inhibition cannot be explained by a disruption in autophagic flux but instead results from genuine induction of cellular autophagy in these systems.

The peptidic pan-caspase inhibitor Z-VAD-fmk is commonly employed as a biochemical tool to probe caspase-mediated apoptosis processes and its ability to induce autophagy in cells had initially been linked to its caspase inhibition effects. However, akin to many small molecule chemical tools, it exhibits a spectrum of effects affecting biological targets other than its primary target. In addition to its caspase inhibition effects, Z-VAD-fmk had previously also been identified as an inhibitor of NGLY1. From a chemical perspective it is not an immediately intuitive NGLY1 inhibitor as it bears little structural resemblance to the natural glycan substrates of the enzyme. Rather, it mimics more closely the peptidic portion of the glycoprotein substrate and has been demonstrated to associate with the peptide binding moiety of the enzyme. Other functionally similar caspase inhibitors do not exhibit these off-target effects. Our study has shown that, unlike Z-VAD-fmk, treatment of cells with the alternative pan-caspase inhibitor Q-VD-OPh does not result in the induction of autophagy. Although Q-VD-OPh is also a peptidic caspase inhibitor and exhibits a similar pan-caspase inhibition profile to Z-VAD-fmk, Q-VD-OPh does not inhibit NGLY1. The use of Q-VD-OPh as a negative control in this study suggests that the induction of cellular autophagy is indeed mediated by NGLY1 inhibition and is unrelated to the inhibition of the cellular caspase targets common to both chemical inhibitors.

Assays for ER stress induction showed that neither the inhibitor treatments (Z-VAD-fmk or Q-VD-OPh) nor siRNA-mediated NGLY1 knockdown result in the induction of two key markers traditionally attributed to cellular stress: HSPA5/BiP (heat shock protein family (Hsp70) member 5) and DDIT3/CHOP (DNA damage inducible transcript 3). It was also noted that there is no evidence of redox imbalance upon either pharmacological or genetic ablation of NGLY1 activity. This result is suggestive that inhibition of NGLY1 by either method is sufficient to trigger the induction of autophagy via mechanisms independent of endoplasmic reticulum stress stimuli and ROS production and is suggestive of crosstalk between the protein quality control mechanisms of ER-associated degradation and autophagic degradation mediated at least in part by NGLY1. Although further investigations will be needed to determine the mechanistic basis of this interaction, it is noted that we observed a transient increase in Thioflavin T fluorescence following treatment with Z-VAD-fmk but not Q-VD-OPh. Such results may be indicative that the accumulation of proteins earmarked for degradation plays a part in autophagy induction. Cells deficient for ATG13 also demonstrate reduced tolerance to both chemical and genetic ablation of NGLY1, suggestive that autophagy may represent an adaptative response to reduced NGLY1 activity. A schematic of the consequences of inhibition of NGLY1 is shown in Figure 1.

**Figure 1.**
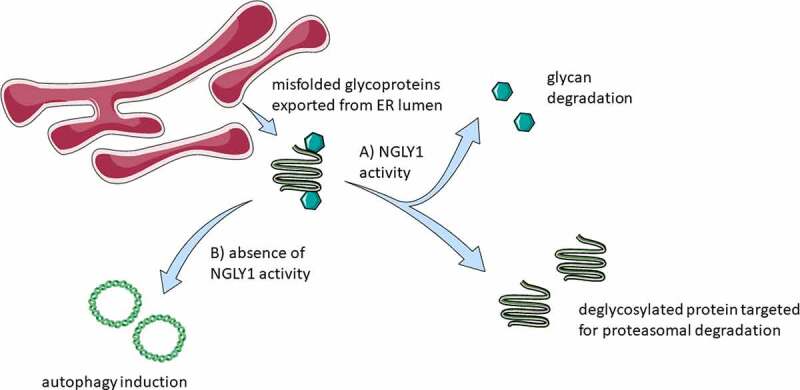
Schematic of consequences of NGLY1 function. Terminally misfolded proteins are exported to the cytosol from the ER lumen. (a) In the presence of NGLY1, glycans are cleaved from the exported proteins and the protein material targeted for degradation via the proteasome. (b) Autophagy induction is observed upon the inhibition of NGLY1. Figure 1 was partly generated using Servier Medical Art, provided by Servier, licensed under a Creative Commons Attribution 3.0 unported license https://creativecommons.org/licenses/by/3.0/

The results presented by this study have multiple implications. Firstly, studies of caspase inhibition which employ the small molecule inhibitor Z-VAD-fmk as a tool are likely to also result in the induction of cellular autophagy. Off-target autophagy induction can be avoided if the alternative caspase inhibitor Q-VD-OPh is utilized in place of Z-VAD-fmk, while maintaining a similar caspase inhibition profile. Regardless, this study acts as a cautionary reminder to consider the specificity and impact of potential off-target effects when designing studies to probe mechanistic aspects of biological pathways, particularly when employing chemical inhibitors.

Secondly, from a biochemical perspective, Z-VAD-fmk has been employed for the induction of autophagic cell death and necroptosis. Inhibition of caspases by the inhibitor blocks induction of apoptosis, while it is important to consider that the induction of autophagy could indeed be mediated by NGLY1 inhibition. In addition, despite its clinical significance and biochemical importance, few specific pharmacological inhibitors of NGLY1 have been identified or developed.

Thirdly, this study also highlights the need of further investigation to determine the extent perturbations in the induction of autophagy plays in the clinical features of NGLY1 deficiency. The variation in clinical presentation and differing severity of symptoms between individual patients coupled with the lack of suitable serum screening techniques akin to those commonly employed for other congenital glycosylation disorders presents significant diagnostic challenges for NGLY1 disorder in a therapeutic setting. It remains to be determined if autophagy-related markers have value as a diagnostic tool or if amelioration of the observed upregulation of cellular autophagy upon NGLY1 inhibition represents an approach which can be exploited for therapeutic intervention.
